# Insights into Genetic and Epigenetic Determinants with Impact on Vitamin D Signaling and Cancer Association Studies: The Case of Thyroid Cancer

**DOI:** 10.3389/fonc.2014.00309

**Published:** 2014-11-04

**Authors:** Grégoire B. Morand, Sabrina Daniela da Silva, Michael P. Hier, Moulay A. Alaoui-Jamali

**Affiliations:** ^1^Department of Otolaryngology-Head and Neck Surgery, Sir Mortimer B. Davis-Jewish General Hospital, McGill University, Montreal, QC, Canada; ^2^Departments of Medicine and Oncology, Segal Cancer Centre and Lady Davis Institute for Medical Research, Sir Mortimer B. Davis-Jewish General Hospital, McGill University, Montreal, QC, Canada

**Keywords:** thyroid cancer, vitamin D, VDR, genome-wide studies, cancer susceptibility

## Abstract

Vitamin D is a key regulator of calcium metabolism and has been implicated as a cancer preventive agent. However, clinical studies have revealed conflicting results on its cancer preventive properties, attributed in part to multiple metabolic and regulatory factors susceptible to affect individual responses to exogenous vitamin D. Vitamin D is obtained from dietary sources and sun exposure, which depends on numerous parameters such as skin type, latitude, and lifestyle factors. Focusing on thyroid cancer (TC), we document that genetic and epigenetic determinants can greatly impact individual response to vitamin D and may outweigh the classical clinical correlative studies that focus on sun exposure/dietary intake factors. In particular, genetic determinants innate to host intrinsic metabolic pathways such as highly polymorphic cytochromes P450s responsible for the metabolic activation of vitamin D are expressed in many organs, including the thyroid gland and can impact vitamin D interaction with its nuclear receptor (VDR) in thyroid tissue. Moreover, downstream regulatory pathways in vitamin D signaling as well as VDR are also subject to wide genetic variability among human populations as shown by genome-wide studies. These genetic variations in multiple components of vitamin D pathways are critical determinants for the revaluation of the potential preventive and anticancer properties of vitamin D in TC.

## Introduction

Thyroid cancer (TC) is the most common endocrine cancer malignancy worldwide (1) with a rising incidence in particular among young patients and women (2–4). Overdiagnosis of subclinical disease, previously proposed as a contributor for the rising incidence, cannot explain the full extent of the increase (5, 6). Risk factors such as exposure to ionizing radiation (7–10), chemical genotoxins (11–13), and obesity (14–17), as well as a lack of protective factors, such as vitamin D deficiency have been implicated in TC increased incidence (18–21).

Vitamin D, an active ingredient of cod-liver oil, was first identified as a cure for rickets in the nineteenth century and has emerged as a principal regulator of calcium homeostasis (22). Cutaneous exposure to sun and dietary intake are the two main natural sources of vitamin D. Vitamin D activity depends on metabolic activation through hydroxylation of the 25 followed by the 1 position of the molecule by cytochromes P450s, which generate the biologically active metabolite 1,25(OH)_2_D3. The action of vitamin D occurs mainly through its binding to the nuclear vitamin D receptor (VDR), which acts as a hormone-regulated transcription factor (23). Upon activation, the VDR forms a heterodimer with related retinoid-X receptors and binds to vitamin D response elements (VDREs) on chromatin regions resulting in the regulation of the expression of several target genes (24–26). VDRE binding by the VDR provides the principle mechanism by which the receptor can activate gene transcription. However, the hormone-bound receptor can also repress gene transcription by a variety of mechanisms (27). Downstream targets of the receptor are involved in mineral metabolism, but VDR also regulates a variety of other metabolic pathways, many of which are components of immune response and cancer signaling (28, 29).

Independent studies support that circulating levels of vitamin D are inversely correlated to several malignancies, including colorectal cancer (30, 31), prostate cancer (32), breast cancer (33, 34), and head and neck squamous cell carcinoma (35, 36). As well, a more recent meta-analysis reported a correlation between vitamin D deficiency and poorer prognosis in several tumor types (37). In TC, several studies point toward a role for impaired 1,25(OH)_2_D3-VDR signaling in the occurrence and progression of the disease (38). This review addresses new insights into genetic and epigenetic determinants of vitamin D response in relation to cancer risk focusing on TC. We provide a systematic review and analysis of experimental and clinical data and the impact of genome-wide analyses on individual susceptibility to TC.

## Materials and Methods

### Genomic database

The UCSC Cancer Genomics Browser (39), a set of web-based tools to display, was used to investigate and analyze cancer genomics data and its clinical information associated with VDR. The browser provides whole-genome to base-pair level views of several different types of genomic data, including next-generation sequencing platforms. Biological pathways, collections of genes, genomic or clinical information were used to sort, aggregate, and zoom into a group of samples. The current release (2013) displays an expanding set of data from various sources, including 201 datasets from 22 The Cancer Genome Atlas (TCGA) cancers as well as data from Cancer Cell Line Encyclopedia and Stand Up To Cancer (39).

### Database of somatic mutations

To collect data on TC related to VDR mutation, the web-software BioMart Central Portal and the Catalog of Somatic Mutations in Cancer (COSMIC) database (40) were used. BioMart offers a one-stop shop solution to access a wide array of biological databases, such as the major biomolecular sequence, pathway, and annotation databases such as Ensembl, Uniprot, Reactome, HGNC, Wormbase, and PRIDE (41). The Cancer BioMart web-interface with the following criteria was used: (1) Primary site = “thyroid”; (2) Mutation ID is not empty. The first criterion ensures that the mutation occurs in thyroid tissues, and the second criterion helps to exclude the samples without mutation in a specific gene. Thereby, we obtained the list of mutations in TC.

Catalog of Somatic Mutations in Cancer (40) stores and displays somatic mutation information and related details on human cancers. COSMIC was developed, and is currently maintained, at the Welcome Trust Sanger Institute. It is designed to gather, curate, and organize information on somatic mutations in cancer and to make it freely available on-line. It combines cancer mutation data, manually curate from the scientific literature, with the output from the Cancer Genome Project (CGP). Genes are selected for full literature curation using the Cancer Gene Census. The current release (v64) describes over 913,166 coding mutations of 24,394 genes from almost 847,698 tumor samples. All genes selected for the COSMIC database came from studies in the literature and are somatically mutated in human cancer (42). Based on this authority resource, a dataset of TC mutation was constructed.

### Data extraction

Information was carefully extracted from all eligible publications including clinical and experimental studies assessing any relation between vitamin D and non-medullary TC. A search for studies in the electronic databases Ovid Medline, Ovid Embase, Web of Science, AMED, and the Cochrane Library was run using an elaborated search strategy (Supplemental Material). In order not to miss any appropriate study, no time or language limits were applied for the search. Review articles were included only temporarily to provide a manual search tool.

The selection of studies involved an initial screening of the title and the abstract. In doubtful cases, the full text was obtained. Articles were entered in the data management software and the duplicates were eliminated (Endnote 6^®^, Thomson Reuters Inc.). For clinical studies, detailed information about participants (number of patients, study location(s), and demographics variables), exposure (sun irradiation, dietary intake, and vitamin D serum level), comparison group, and outcome was assessed.

**Figure 1:**
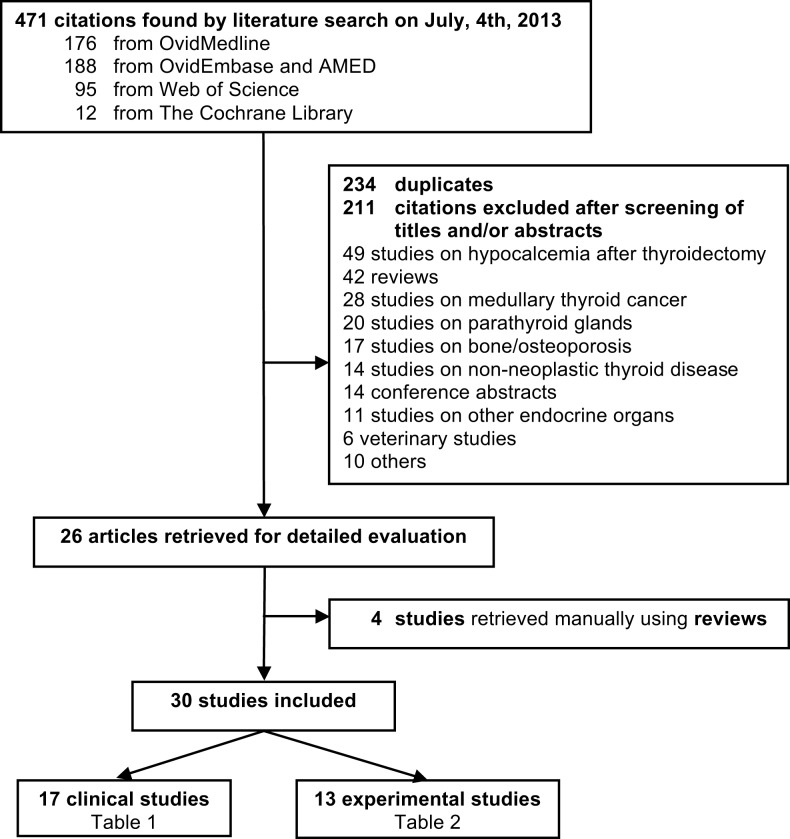
**Flow chart of study selection for systematic review**.

The search retrieved 471 references published until July 4th, 2013, 12 from the Cochrane Library, 176 from Ovid Medline, 188 from Ovid Embase and AMED, and 95 from Web of Sciences. Crosschecking the references of the reviews led to the inclusion of four supplementary articles (43–46). No clinical trial was available. The flow chart of study selection is shown in Figure 1.

Overall 30 articles were included, of which 17 were clinical studies (Table 1) and 13 experimental studies (Table 2). These studies were published in English language from 1987 to 2013. Of the 17 clinical studies, 8 (47.0%) showed protective effect of vitamin D (44, 45, 47–52), 6 (35.3%) no significant relationship (43, 46, 53–57), and 2 (11.7%) revealed an increased TC risk with high vitamin D intake (58, 59). No comparison could be drawn from the remaining study (5.8%) (60). TC incidence was assessed in all of these studies, mortality in two (45, 47); and one report assessed both (45). Except for three studies involving Arab populations (51, 56, 60), all studies included Europeans’ descendants and/or Hispanic whites.

**Table 1:** **Summary of clinical studies reporting an association between thyroid cancer and vitamin D**.

First author	Pub year	Country (state/province)^a^	Cases/controls	Outcome	Exposure	Results^b^
Akslen (44)	1998	Norway	2627/NA	Incidence	Seasonal variation	pro
Boscoe (45)	2006	USA	>4,000/>4,000	Incidence and mortality	Latitude	pro^c^
D’avanzo (53)	1997	Italy	399/617	Incidence	Intake	NS
Glattre (54)	1993	Norway	92/460	Incidence	Intake	NS
Grant (47)	2006	Spain	NR	Mortality	Latitude	pro
Greenlee (58)	2004	USA (WA)	305/64,226	Incidence	Intake	con
Haghpanah (56)	2007	Iran	71/82	Incidence	VDR polymorphism	NS
Jonklass (46)	2013	USA (DC)	48/17	Incidence	Serum 25(OH)D	NS
Laney (55)	2010	USA (NE)	24/42	Incidence	Serum 25(OH)D	NS
Mack (43)	2002	USA (CA)	292/292	Incidence	Intake	NS
Penna-Martinez (48)	2009	Germany	147/57	Incidence	Serum 1,25(OH)_2_ D VDR Polymorphism	pro
Penna-Martinez (49)	2012	Germany	253/302	Incidence	Serum 1,25(OH)_2_ D	pro
Peterson (60)	2011	USA (MI)	30/70	NA	Sun exposure	NA
Ron (59)	1987	USA (CT)	159/285	Incidence	Intake	con
Roskies (50)	2012	Canada (QC)	12/88	Incidence	Serum 25(OH)D	pro
Sahin (51)	2013	Turkey	344/116	Incidence	Serum 25(OH)D	pro
Stepien (52)	2010	Poland	50/26	Incidence	Serum 1,25(OH)_2_ D	pro

^a^WA, Washington; DC, District of Columbia; NE, Nebraska; CA, California; MI, Michigan; CT, Connecticut; QC, Quebec;

^b^pro, protective effect of vitamin D (or surrogates); NS, not significant; con, vitamin D (or surrogates) increasing risk; NA, not applicable;

*^c^for women only*.

**Table 2:** **Experimental studies using cell lines or preclinical models to assess vitamin D effect on thyroid cancer**.

First author	Pub year	Samples^a^	Main results
Balla (61)	2011	6 PTC	Overexpression of CYP24A1 mRNA
Bennett (62)	2012	TPC1, C643	Antiproliferative effect of calcitriol
Clinckspoor (63)	2011	FTC133, C643, 8505c, Hth74	Antiproliferative effect of calcitriol and superagonistic analog CD578
Clinckspoor (64)	2012	64 thyroid cancers	VDR, CYP24A1, CYP27B1 overexpression
Dackiw (65)	2004	15 SCID mice/WRO	Growth inhibition of orthotopic tumor and p27^kip1^ restoration after calcitriol treatment
Khadzkou (66)	2006	44 PTC	Overexpression of VDR and CYP27B1 (FFPE)
Liu (67)	2002	NPA, WRO	Antiproliferative effect of calcitriol and superagonistic analog EB1089, p27 restoration
Liu (68)	2005	WRO	Calcitriol and its analog EB1089 restore PTEN-dependent fibronectin expression
		SCID mice/WRO	Growth inhibition in heterotopic model with calcitriol and EB1089
Liu (69)	2011	WRO, MRO	Calcitriol inhibits CEACAM1
Okano (70)	1999	Nude mice/NPA	Trend to growth inhibition in heterotopic model with calcitriol and less-calcemic analog
		NPA	Dose-dependent inhibition of calcitriol and less-calcemic analog
Sharma (71)	2010	TPC1, C643, Hth7, Hth74, 8505c, SW1736	Response to calcitriol/DP006 depending on VDR polymorphism and 24-hydroxylase levels
Somjen (72)	2013	NPA, ARO, MRO	Overexpression of VDR and CYP27B1
Suzuki (73)	1999	TPC1-4, TAC1, TTA1	Dose-dependent growth inhibition of calcitriol and less-calcemic analog

*^a^Cell line-corresponding histologic subtype: TPC1-4-PTC, KTC-PTC, BCPAP-PTC, NPA-PTC, KAT5-PTC, FTC133-FTC, FRO-FTC, MRO-FTC, WRO-FTC, C643-ATC, Hth7-ATC, Hth74-ATC, 8505c-ATC, SW1736-ATC, TAC-1-ATC, TTA-1-ATC. PTC, papillary thyroid cancer; FTC, follicular thyroid cancer; ATC, anaplastic thyroid cancer; SCID, severe combined immunodeficient*.

## Results and Discussion

### Determinants of vitamin D levels and impact in TC

Solar UVB irradiation is the primary source of vitamin D and can be estimated by latitude of the living area. In TC, large epidemiological studies support an inverse relation between TC incidence and latitude (45, 47) (Table 1). These studies performed a multivariate analysis to adjust for confounding factors. However, vitamin D levels were not measured. Consequently, it is unclear if the multivariate analysis resulted in accurate vitamin D estimates. Indeed, vitamin D deficiency is highly prevalent among latitudes that benefit from high solar irradiation such as Africa, the Middle East, and Southern Asia. This may be due to skin pigmentation, traditional clothing, and sun avoidance seen in southern heat-exposed populations (60, 74). In contrast, fair-skinned northern populations usually seek sun exposure and may also benefit from high intake of vitamin D rich diet such as fatty fish and cod-liver oil (74). Further, a mutation in the cutaneous structural protein filaggrin, which occurs in up to 10% of Europeans was shown to lead to higher circulating vitamin D levels (75). Nonetheless, North American and European studies have shown seasonal variations of vitamin D levels due to insufficient sun irradiation during winter (76). In TC, one study from Norway reported higher proliferation values for tumors resected during winter compared to other seasons (44). These results comply with above-mentioned studies showing an inverse relation between TC incidence and latitude (45, 47). For studies estimating vitamin D consumption and TC risk, however, no convincing associations have been shown (Table 1) (43, 53, 54, 58, 59). This may be due to the general poor correlation between vitamin D deficiency and estimates of vitamin D consumption (57).

A more accurate way to assess vitamin D is biological monitoring. Association studies investigating the relationship between levels of serum vitamin D and TC risk mostly point toward a protective effect of vitamin D (48–52, 55, 77) (Table 1). Pooling the data among these studies is not possible due to different cut-off levels for different vitamin D derivatives and control groups used in each of these studies. This would greatly limit the validity of a meta-analysis. The lack of consensus in cut-off levels may reflect the fact that those are differently defined depending on targeted clinical endpoints (78, 79). Classical vitamin D targets, i.e., those implicated in calcium and bone homeostasis, do not allow conclusions on optimal level of vitamin D having anticancer properties. While doses up to 4,000 IU of daily vitamin D supplementation have been considered safe, studies have reported hypercalcemia, nephrolithiasis, vascular, and soft tissue calcification with high doses of vitamin D and also U-shaped relationship between vitamin D levels above 75 nmol/l and certain cancer subtypes (80, 81). One additional issue of most of these association studies is that vitamin D levels were measured only once, which does not permit distinction between outcome and exposure. Indeed, some studies have reported low serum vitamin D as a result of malignancy (82).

Above-mentioned skin types, alimentary, and social habits yet do not fully explain vitamin D variability among populations (83). One major determinant of individual susceptibility to vitamin D is the activity of vitamin D metabolizing enzymes. Three major cytochrome P-450 (CYP) hydroxylases are responsible for vitamin D activation through 25- followed by 1α-hydroxylation of the molecule, and deactivation through 24-hydroxylation. Multiple enzymes have been reported as vitamin D 25-hydroxylases, a step occurring constitutively and primarily in the liver. Unlike 25-hydroxylation, 1α-hydroxylation of 25(OH)D_3_ by the CYP27B1 is a tightly regulated and rate-limiting step. It is regulated by calcium, 1α,25(OH)_2_D_3_ itself, PTH, calcitonin, and phosphate levels. Recently, fibroblast growth factor 23 (FGF23) was identified as a novel antagonist of PTH and is thought to play an important role in vitamin D regulation pathway (84). Although CYP27B1 and CYP24A1 are primarily expressed in the kidney, recent studies showed that they are expressed in many other tissues, including the thyroid (61, 62). In TC, there is evidence that polymorphisms leading to impaired CYP27B1 function and/or increased CYP24A1 activity are associated with increased TC risk (49). Transcriptional profiling studies show that both enzymes are overexpressed in early TC (61), but their expression tends to decrease along with tumor progression (64, 66).

### Determinants of predicted response to vitamin D

**Figure 2:**
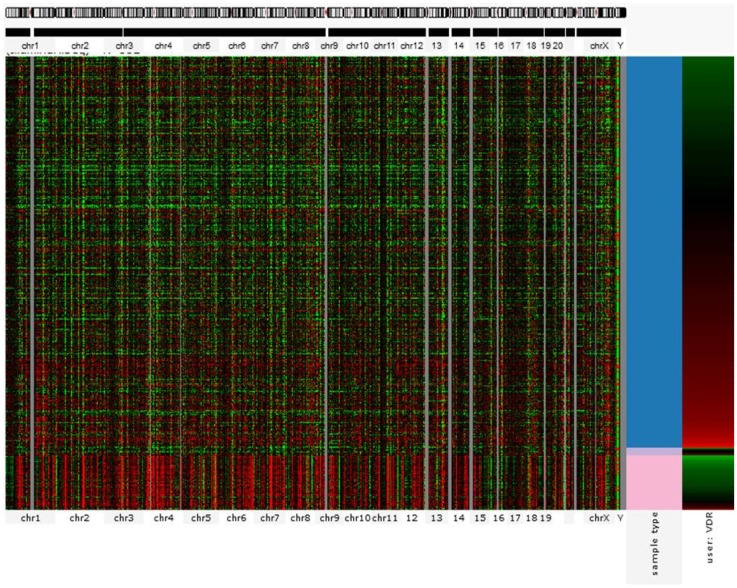
**Gene expression profile showing VDR signature for 552 thyroid cancer cases (RNA Seq)**. Each row corresponds to sample from a single case. Columns from the left correspond to genomic heatmap according to chromosomal location. The last two columns represent VDR expression profile (represented by red for overexpression and green for downregulation) in normal (pink) versus cancer (red) tissues. VDR is mostly overexpressed in malignant samples but almost absent in benign tissues. Source: UC Santa Cruz – Cancer Genomics Browser.

**Figure 3:**
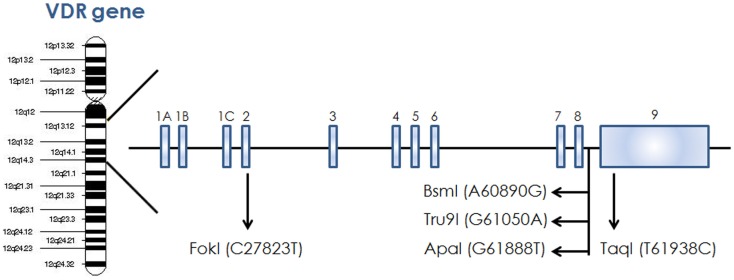
**Schematic diagram of VDR gene showing different restriction site on chromosome 12**.

**Figure 4:**
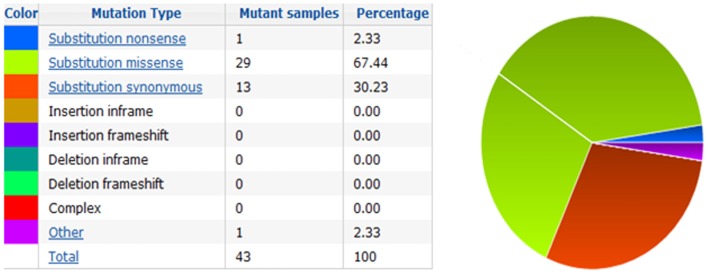
**Pie-chart showing the percentage of the mutation type in VDR in thyroid cancer according to COSMIC database**.

The action of vitamin D mainly occurs through binding to the VDR (23), whose levels are subject to genetic variations. Using the UCSC genomic database, we analyzed 552 thyroid samples that underwent genomic profiling using RNA Seq. The expression of VDR was down regulated in benign thyroid samples and up regulated in most TC cases (Figure 2). These results are confirmed by a few *in vitro* studies using TC cell lines (72) and independent clinical samples (64, 66). However, VDR levels alone may translate poorly with response to vitamin D stimulation if polymorphisms of VDR are not taken into account (71, 85, 86). The analysis of the genomic organization of the VDR *locus* at chromosome 12q13.1 revealed the large *VDR* gene (about 100 Kb) with an extensive promoter region capable of generating multiple tissue-specific transcripts (87). In view of the observed genome-wide frequency of single nucleotide polymorphisms (88), one can predict >100 functional polymorphisms to be present in the VDR region alone, including the promoter region (Figure 3). Point mutations in the VDR gene have been identified in various regions, including the VDR DNA binding domain (DBD) and the ligand-binding domain (LBD) (89). Such mutations can disrupt ligand-binding affinity to the receptor (90), heterodimerization of VDR with RXR (91), or interactions of the VDR receptor with partners such as coactivators (92). Other mutations such as in the initiation codon can create a premature termination (93) or alternative translation start sites to result in alternative splicing and formation of truncated proteins (94, 95). The analysis of the COSMIC database showed a high proportion of missense mutations that were re-identified (67.44%), while complex mutations were not detected (Table 3). The distribution of the mutations observed in the VDR gene in TC is shown in Figure 4. Only two studies investigated the association between VDR polymorphisms and TC risk, one showed an increased TC risk for patients with particular VDR polymorphism (48), while another could not point out any significant difference (56).

**Table 3:** **Mutations identified in VDR**.

Position (AA)	Mutation (CDS)	Mutation (amino acid)	Mutation type
8	c.23C > T	p.T8I	Substitution – missense
33	c.98G > A	p.G33D	Substitution – missense
52	c.156G > A	p.M52I	Substitution – missense
74	c.221G > A	p.R74H	Substitution – missense
78	c.233C > G	p.A78G	Substitution – missense
130	c.389G > A	p.R130H	Substitution – missense
146	c.438C > G	p.T146T	Substitution – coding silent
149	c.445G > T	p.D149Y	Substitution – missense
154	c.460C > T	p.R154W	Substitution – missense
158	c.472C > T	p.R158C	Substitution – missense
159	c.477G > C	p.V159V	Substitution – coding silent
161	c.481G > A	p.D161N	Substitution – missense
162	c.484G > T	p.G162C	Substitution – missense
169	c.507G > A	p.R169R	Substitution – coding silent
181	c.541G > T	p.D181Y	Substitution – missense
191	c.573C > A	p.I191I	Substitution – coding silent
199	c.597G > A	p.S199S	Substitution – coding silent
208	c.623G > T	p.S208I	Substitution – missense
236	c.708C > A	p.Y236^a^	Substitution – nonsense
253	c.757G > T	p.D253Y	Substitution – missense
274	c.820C > T	p.R274C	Substitution – missense
296	c.887G > A	p.R296H	Substitution – missense
320	c.960G > A	p.L320L	Substitution – coding silent
339	c.1015G > A	p.V339I	Substitution – missense
350	c.1049C > T	p.A350V	Substitution – missense
350	c.1050G > A	p.A350A	Substitution – coding silent
352	c.1056T > C	p.I352I	Substitution – coding silent
353	c.1058A > T	p.E353V	Substitution – missense
358	c.1072C > T	p.R358C	Substitution – missense
365	c.1094C > T	p.T365M	Substitution – missense
368	c.1103G > A	p.R368H	Substitution – missense
379	c.1135C > T	p.L379F	Substitution – missense
399	c.1196A > T	p.K399M	Substitution – missense
402	c.1205G > C	p.R402P	Substitution – missense
418	c.1254G > T	p.V418V	Substitution – coding silent
420	c.1258G > A	p.E420K	Substitution – missense

*^a^Nonsense mutation resulting in stop codon*.

### Downstream impact of VDR activation

Upon activation by vitamin D, VDR binds as a heterodimer with retinoid-X receptors to specific VDREs (84). VDREs usually bear a consensus sequence known as DR3 element located in the promoter region of the target genes. In addition to this classic mechanism, recent chromatin-immunoprecipitation (ChIP-seq) studies allowed to gain genome-wide insights of the binding sites of VDR (96). These studies showed that the ligand-bound heterodimer can bind to ~2000–8000 sites in the genome. Interestingly, the majority of the binding sites do not bear the classical DR3-type sequence (84). A significant enrichment was seen in regions associated with active chromatin and histone modifications thus supporting a broad genetic and epigenetic regulatory role of vitamin D. Further enrichment of VDR binding was also found in proximity of genes involved in autoimmune diseases (e.g., multiple sclerosis, type-I diabetes, and Crohn’s disease) and colorectal or breast cancer (97). For TC, only data relying on classical *in vitro* experiments is available.

In agreement with experimental studies in other cancer types, exposure of a variety of TC cells to vitamin D *in vitro* leads to antiproliferative and pro-differentiation properties (62, 63, 67, 70, 71, 73) (Table 2). These results have been confirmed by *in vivo* studies (65, 68). Most studies are testing vitamin D itself and synthetic vitamin D analogs, as patient’s exposure to pharmacologically high doses of vitamin D can be limited by the side-effects, mainly hypercalcemia (63, 67, 70, 71, 73).

Mechanistically, vitamin D was shown to inhibit proliferation through c-mac mRNA inhibition, which is a well-known proto-oncogene (70). Further, it can induce a growth arrest effect in part through stimulating accumulation of the cyclin-dependent kinase inhibitor p27^kip1^ in the nucleus (67). Treatment with vitamin D is thought to prevent p27^kip1^ phosphorylation, which was shown to increase its ubiquitin-dependent proteasome degradation (67). Further, vitamin D was shown to enhance cell–cell adhesion through PTEN-dependent fibronectin upregulation (68). Those results could be confirmed *in vivo*. Interestingly, the antiproliferative effect of vitamin D was abolished when knocking down fibronectin (68) and was shown to be independent of CEACAM1 expression, a tumor-suppressive adhesion molecule (69).

## Conclusion and Perspectives

The pleiotropic roles of vitamin D in cancer have been recognized through seminal preclinical studies although the preventive and therapeutic potential of vitamin D or its analogs remain debated due in part to the complex mode of action of this vitamin. Recent progress in high-throughput technologies to interrogate human genomic and epigenomic events has provided additional levels of regulatory loops and individual genetic variations that can impact on individual susceptibility to vitamin D. This knowledge opens up new tools to address confounding factors that contribute to discrepant results seen in previous association studies, in particular in relation to cancer prevention. As well, this knowledge impels an exciting avenue in the discovery of novel vitamin D analogs with enhanced preventive or therapeutic efficiency and limited side-effects.

## Author Contributions

Gregoire B. Morand performed the literature search, the retrieval of the studies, the data extraction, and wrote the main part of the manuscript under Sabrina Daniela da Silva and Moulay A. Alaoui-Jamali’s supervision. All the authors participated substantially to the final manuscript and approved the final version.

## Conflict of Interest Statement

The authors declare that the research was conducted in the absence of any commercial or financial relationships that could be construed as a potential conflict of interest.

## Supplementary Material

The Supplementary Material for this article can be found online at http://www.frontiersin.org/Journal/10.3389/fonc.2014.00309/abstract
