# Supplementary material for: Insights into Genetic and Epigenetic Determinants with Impact on Vitamin D Signaling and Cancer Association Studies: The Case of Thyroid Cancer
Source: Front Oncol. 2014 Nov 4;4:309. doi: 10.3389/fonc.2014.00309 (PMC4220101; doi:10.3389/fonc.2014.00309)
Supplement: Supplementary file 1 [file Data_Sheet_1.DOCX]

**Supplemental material**: search strategy used for systematic review

1. exp Vitamin D/

2. exp Receptors, Calcitriol/

3. Calcitriol/

4. Vitamin D Deficiency/

5. calcitriol*.tw.

6. vitamin D*.tw.

7. cholecalciferol*.tw.

8. ergocalciferol*.tw.

9. "1,25(OH)2 D3".tw.

10. 1 or 2 or 3 or 4 or 5 or 6 or 7 or 8 or 9

11. exp Thyroid Neoplasms/

12. thyroid cancer*.tw.

13. thyroid neoplasm*.tw.

14. thyroid carcinoma*.tw.

15. thyroid tumo*.tw.

16. 11 or 12 or 13 or 14 or 15

17. 10 and 16
